# Correction of scan time dependence of standard uptake values in oncological PET

**DOI:** 10.1186/2191-219X-4-18

**Published:** 2014-04-03

**Authors:** Jörg van den Hoff, Alexandr Lougovski, Georg Schramm, Jens Maus, Liane Oehme, Jan Petr, Bettina Beuthien-Baumann, Jörg Kotzerke, Frank Hofheinz

**Affiliations:** 1 PET Center, Institute of Radiopharmaceutical Cancer Research, Helmholtz-Zentrum Dresden-Rossendorf, Dresden 01328, Germany; 2 Department of Nuclear Medicine, University Hospital Carl Gustav Carus, Technische Universität Dresden, Dresden 01307, Germany; 3 Faculty of Medicine, Technische Universität Dresden, Dresden 01307, Germany

**Keywords:** SUV, SUR, Tumor-to-blood ratio, PET, Time dependence, Uptake period, FDG, Dual time point

## Abstract

**Background:**

Standard uptake values (SUV) as well as tumor-to-blood standard uptake ratios (SUR) measured with [ ^18^F-]fluorodeoxyglucose (FDG) PET are time dependent. This poses a serious problem for reliable quantification since variability of scan start time relative to the time of injection is a persistent issue in clinical oncological Positron emission tomography (PET). In this work, we present a method for scan time correction of, both, SUR and SUV.

**Methods:**

Assuming irreversible FDG kinetics, SUR is linearly correlated to *K*_m_ (the metabolic rate of FDG), where the slope only depends on the shape of the arterial input function (AIF) and on scan time. Considering the approximately invariant shape of the AIF, this slope (the ‘Patlak time’) is an investigation independent function of scan time. This fact can be used to map SUR and SUV values from different investigations to a common time point for quantitative comparison. Additionally, it turns out that modelling the invariant AIF shape by an inverse power law is possible which further simplifies the correction procedure. The procedure was evaluated in 15 fully dynamic investigations of liver metastases from colorectal cancer and 10 dual time point (DTP) measurements. From each dynamic study, three ‘static scans’ at *T*=20,35,and 55 min post injection (p.i.) were created, where the last scan defined the reference time point to which the uptake values measured in the other two were corrected. The corrected uptake values were then compared to those actually measured at the reference time. For the DTP studies, the first scan (acquired at (78.1 ± 15.9) min p.i.) served as the reference, and the uptake values from the second scan (acquired (39.2 ± 9.9) min later) were corrected accordingly and compared to the reference.

**Results:**

For the dynamic data, the observed difference between uncorrected values and values at reference time was (-52±4.5)% at *T*=20 min and (-31±3.7)% at *T*=35 min for SUR and (-30±6.6)% at *T*=20 min and (-16±4)% at *T*=35 min for SUV. After correction, the difference was reduced to (-2.9±6.6)% at *T*=20 min and (-2.7±5)% at *T*=35 min for SUR and (1.9% ± 6.2)% at *T*=20 min and (1.7 ± 3.3)% at *T*=35 min for SUV. For the DTP studies, the observed differences of SUR and SUV between late and early scans were (48 ± 11)% and (24 ± 8.4)%, respectively. After correction, these differences were reduced to (2.6 ± 6.9)% and (-2.4±7.3)%, respectively.

**Conclusion:**

If FDG kinetics is irreversible in the targeted tissue, correction of SUV and SUR for scan time variability is possible with good accuracy. The correction distinctly improves comparability of lesion uptake values measured at different times post injection.

## Background

Currently, the standard uptake value (SUV, in units of grams per milliliter), defined as the tracer concentration at a certain time point normalized to injected dose per unit body weight, is the only practical means typically used for quantitative evaluation of clinical [ ^18^F-]fluorodeoxyglucose (FDG) positron emission tomography (PET) investigations. However, the SUV approach has several known shortcomings [1-3] which affect the reliability of the SUV as a surrogate of the metabolic rate of glucose consumption. Among these are 

1. Susceptibility to errors in scanner calibration,

2. Insufficient correlation between systemic distribution volume and body weight (leading to variants of the SUV approach using lean body mass (SUV _*lbm*_) [4] or body surface area (SUV _*bsa*_) [5] for normalization), and, consequentially, residual inter-study variability of the arterial input function (AIF) despite SUV normalization [6].

3. Time dependence of the SUV.

We have addressed the first two points in a recent publication [7] and demonstrated that the standard tumor-to-blood uptake ratio (SUR) is superior to SUV as a surrogate parameter of *K*_m_ (the metabolic rate of FDG). The reason for this is that SUR can be shown to be linearly related to *K*_m_ if the AIF exhibits an essentially invariant shape across different investigations. Scale changes of the AIF (different blood SUVs) do not have any influence on SUR, whereas lesion SUV is directly affected by the latter.

While tumor SUR thus is not affected by inter-study variability of blood SUV, its use does not address another source of potentially serious variability, namely, insufficient standardization of the uptake time prior to scanning. Variability of the uptake period (i.e., variability of scan start time relative to the time of injection) is a persistent issue in clinical oncological PET [8,9]. This represents a well-known problem for meaningful SUV quantification since tumor SUV distinctly increases over time [1,2,10,11]. Especially affected are follow-up studies, where SUV changes of tumor lesions between consecutive studies are the relevant quantity (e.g., in the context of therapy response assessment), and scan time variability can lead to serious misinterpretation of the data.

We are aware of two studies addressing the question how to correct SUVs for variable scan time [10,11]. In [10], a general SUV correction formula is proposed, but the emphasis of this paper lies on considerations regarding optimizing the imaging time, and the proposed semi-empirical correction formula is not fully evaluated. It contains, however, the important insight that the shape of the AIF enters the correction. A second investigation of the scan time variability effect and an empirical formula how to correct for it has been reported in [11]. This investigation was restricted to breast cancer patients, and the proposed correction formula resulted from purely empirical observation of how SUV varied in these patients over time without explicitly considering the influence of the AIF shape. Here, the important observation was made that the rate of SUV change over time is approximately proportional to the magnitude of the SUV itself.

In the present work, we propose a generic method for correction of scan time variability effects on SUR (and SUV) which is based on observation and utilization of the specific shape invariance of FDG input functions and the consequences following from it when analyzing the Patlak equation. Especially, this provides the ability to map all measured values to a common reference scan time (e.g., 60 min p.i.) as long as FDG kinetics can be considered irreversible in the targeted tissue.

Starting from the Patlak equation, we first derive the correction formulas for SUR and SUV, respectively. The correction procedure is then evaluated in a group of nine patients with liver metastases of colorectal cancer, for which 15 dynamical investigations are available. These data enable assessment of the full dynamic AIF as well as comparison of the actually observed tumor SUV changes over time with the correction factors derived for two time frames selected from the dynamic data. Furthermore, we apply the correction procedure to an independent group of ten dual time point (DTP) whole body studies, where the full AIF is not known. Here, the correction is used to map SUR (and SUV) from the second to the first time point to investigate whether the corrected SURs and SUVs are concordant with those determined at the first time point as should be the case if the correction works well.

## Methods

### Theory

According to the Patlak equation [12,13], the lesion SUR at time *t* is given by

(1) $$\text{SUR}\left(t\right)=\frac{c_{t}\left(t\right)}{c_{a}\left(t\right)}=K_{m}\times \Theta \left(t\right)+V_{r},$$

where *c*_t_(*t*) is the lesion’s tracer uptake until time *t*, and *c*_a_(*t*) is the AIF at the same time point. *Θ* (the independent (*x*-) coordinate of the Patlak plot) is determined by the AIF according to

(2) $$\Theta \left(t\right)=\frac{\int_{0}^{t}c_{a}\left(s\right)\text{ds}}{c_{a}\left(t\right)}=\frac{\text{AUC}\left(t\right)}{c_{a}\left(t\right)},$$

which dimensionally is a time (‘Patlak time’). *K*_m_ is the respective lesion’s metabolic trapping rate, and *V*_r_ is the so-called apparent volume of distribution.

As has been discussed in [7], assuming a suitable lesion and investigation independent value ${\bar{V}}_{r}$ for *V*_r_ does not introduce sizable errors since the second term in Equation 1 is in general distinctly smaller than the first one, while its inter-lesion variability is rather small. It was also shown in [7] that to a quite good approximation, the AIF is shape invariant (although differing in scale even after SUV normalization) across investigations so that *Θ*(*t*) no longer depends on the individual AIF but can be considered as a given function of the scan time *t* alone. This offers the possibility of correcting SURs (and SUVs) for scan start time variations as follows.

We consider a reference scan time *T*_0_ which can be taken to be equal to the intended ‘ideal’ mid-scan time, e.g., 60 min p.i.. With a suitable chosen fixed value for ${\bar{V}}_{r}$ and the invariant (investigation and lesion independent) *Θ*(*t*) relation, we can solve Equation 1 for the lesion’s *K*_m_ at both the actually given scan time *t*=*T* and the reference time *t*=*T*_0_. Equating both expressions yields

(3) $$\frac{{\text{SUR}}_{T}-{\bar{V}}_{r}}{\Theta_{T}}=\frac{{\text{SUR}}_{0}-{\bar{V}}_{r}}{\Theta_{0}},$$

where we use indices *T* and 0 to indicate measurements referring to times *T* and *T*_0_, respectively. Solving this equation for SUR_0_ yields

(4) $${\text{SUR}}_{0}=\frac{\Theta_{0}}{\Theta_{T}}\;\left({\text{SUR}}_{T},-,{\bar{V}}_{r}\right)+{\bar{V}}_{r},$$

which determines the required scan time correction, provided the actual functional dependence of *Θ* on *T* is known. As it turns out, for the available 15 dynamic investigations, *Θ*(*T*) is quite well approximated by a straight line through the origin (i.e., proportional to *T*). This is demonstrated in Figure 1B. Figure 1 further demonstrates that inter-individual variability around the average AIF appears small, although it has a non-negligible influence on lesion SUV as discussed in [7].

**Figure 1:**
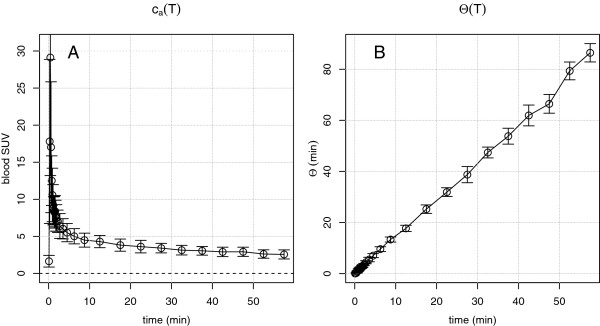
**Group averaged arterial input function (A) and ‘Patlak time’ (B) for the available 15 dynamic liver investigations.** Error bars are point-wise standard deviations of the individual curves from the respective mean (in the case of AIF, this amounts to (23±3)% for *t*>1 min).

In mathematical terms, the approximate proportionality between *T* and *Θ* is equivalent to stating that starting early after the peak of the curve (*t*>1 min), the time dependence of the AIF can be very well described by an inverse power law of the form *c*_a_(*t*)=*A*×(*t*-*d*)^-*b*^, where the position of the singularity of the hyperbola at *t*=*d* is located near the peak position of the curve. While inclusion of *d* as a free parameter generally improves quality of the fit, somewhat, it turns out that it suffices, for the problem at hand, to consider the case *d*=0 and use the model

(5) $$c_{a}\left(t\right)=A\times t^{-b}\;\;\left(t,>,1,\;,\text{min}\right).$$

Figure 2 shows the still quite satisfactory fit of this model to the group averaged data from Figure 1A. Since empirically the condition *b*<1 is always fulfilled, we can extend the fitted hyperbola to *t*=0 and integrate up to some time point *t*=*T* which yields the area under the curve

(6) $$\text{AUC}\left(T\right)=\frac{A}{1-b}\times T^{1-b}$$

**Figure 2:**
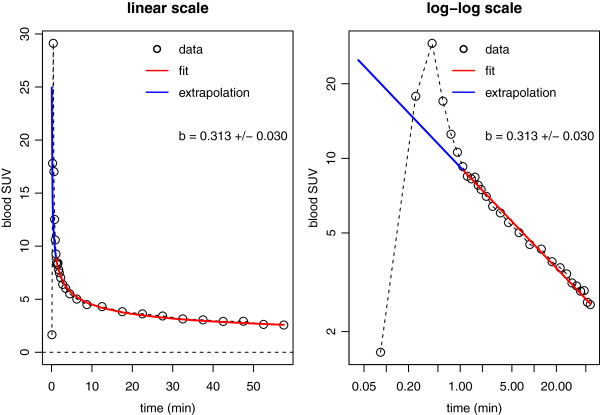
**Least squares fit of hyperbola (Equation **5**) to group-averaged AIF.** The fit is shown in linear scale (left) as well as in double logarithmic scale (right). The specified uncertainty of the time exponent *b* is the standard deviation of this parameter when fitting the model separately to each of the 15 contributing AIFs which indicates the observed inter-individual variability. The data range indicated in red is used for the fit. The blue line extrapolates the fitted hyperbola to early times. Despite the obvious deviation of the hyperbola from the data in this time range, contribution to total AUC is small, and the residual difference negligible if AUC up to times *t*>20 min is considered.

and consequently,

(7) $$\Theta \left(T\right)=\frac{\text{AUC}\left(T\right)}{c_{a}\left(T\right)}=\frac{T}{1-b},$$

which is a straight line through the origin with slope *m*=(1-*b*)^-1^. Equation 7 immediately explains the observed approximate proportionality between *Θ* and *T* and determines the factor of proportionality as being equal to *m*. Taking Equation 7 into account, Equation 4 can be rewritten as

(8) $${\text{SUR}}_{0}=\frac{T_{0}}{T}\;\left({\text{SUR}}_{T},-,{\bar{V}}_{r}\right)+{\bar{V}}_{r}$$

so that the deviation of SUR _*T*_ from SUR _0_ (the ‘error’ due to deviation of actual scan time from the reference time) is given by

(9) $$\begin{array}{c} {\text{SUR}}_{T}-{\text{SUR}}_{0}=\left(1-\frac{T_{0}}{T}\right)\;\left({\text{SUR}}_{T}-{\bar{V}}_{r}\right)\\ \;=\frac{\mathrm{\Delta T}}{T}\left({\text{SUR}}_{T}-{\bar{V}}_{r}\right). \end{array}$$

It is notable that the AIF does not enter Equation 8 explicitly. According to this equation, the ratio SUR_0_/SUR_*T*_ is furthermore roughly equal to the ratio *T*_0_/*T* of the respective scan time points (and thus independent of the value of SUR_*T*_) if ${\bar{V}}_{r}\ll {\text{SUR}}_{T}$. This might be taken as a rule of thumb for estimating the magnitude of the scan time effect on SUR. The SUR correction procedure is illustrated in Figure 3, where the effect of a ±20-min deviation from the chosen reference time *T*_0_=60 min is shown. Ultimately, the correction procedure maps the measured SUR_*T*_ values from the respective straight line SUR_*T*_(*K*_m_) to the straight line SUR_0_(*K*_m_).

**Figure 3:**
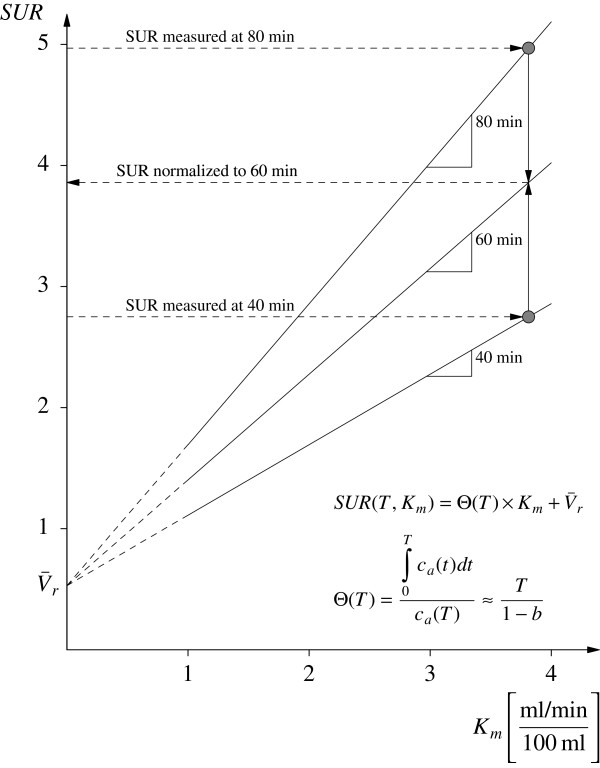
**Schematic explanation of how correction for scan time variability is performed.** The example illustrates the effect of a ±20-min deviation from the targeted scan time (*T*=60 min). The straight lines define the *K*_m_ vs. SUR relation presuming ${\bar{V}}_{r}=0.53$ and the linear *Θ*(*T*) relation according to Equation 7 with *b*=0.313. The correction procedure yields the SUR values which would have been measured if all scans had been performed at *T*=60 min. Without correction, there is a spurious fractional deviation that is roughly equal to the corresponding deviation of the actual from the targeted scan time (1/3 in this example).

Recalling the relation between SUR and SUV (i.e., SUR(*t*)=SUV(*t*)/*c*_a_(*t*) if SUV units are used for expressing the tracer concentrations in tissue and blood, respectively) and taking into account Equation 5, the procedure can also be used to correct the scan time dependence of the SUVs:

(10) $$\begin{array}{l} \frac{{\text{SUV}}_{0}}{{\text{SUV}}_{T}}=\frac{{\text{SUR}}_{0}}{{\text{SUR}}_{T}}\times \frac{c_{a}\left(T_{0}\right)}{c_{a}\left(T\right)}\;\Rightarrow \;\\ {\text{SUV}}_{0}={\text{SUV}}_{T}\times \left[\frac{{\text{SUR}}_{0}}{{\text{SUR}}_{T}}\times {\left(\frac{T_{0}}{T}\right)}^{-b}\right]\;, \end{array}$$

where the SUR_0_/SUR_*T*_ ratio follows from Equation 8. Contrary to the latter equation, Equation 10 exhibits an explicit dependence on the time exponent *b* and thus depends on the rate of decrease of the AIF. Using the rough estimate SUR_0_/SUR_*T*_≈*T*_0_/*T* given above, one now obtains the rule-of-thumb estimate SUV_0_/SUV_*T*_≈(*T*_0_/*T*)^1-*b*^ for the scan time effect on SUV.

### Study sample

#### Dynamic data

Nine male patients with liver metastases of colorectal cancer were included retrospectively (age 48 to 76 years (mean 62.8), weight 73 to 100 kg (mean 85.5)). For each patient, one to three dynamic PET scans of 60-min duration were performed (altogether 15 scans). The scans started immediately after injection of 346 to 430 MBq FDG administrated as bolus over 10 to 20 s.

The scans were conducted with an ECAT EXACT HR ^+^, Siemens/CTI (Knoxville, TN, USA). The acquired data were sorted into 23 to 31 frames with 10- to 20-s duration during bolus passage, 30- to 150-s duration until 10 min p.i., and 300-s duration afterwards. Tomographic images were reconstructed using attenuation weighted OSEM reconstruction (6 iterations, 16 subsets, 6-mm FWHM Gaussian filter). The study protocol was approved by the Technische Universität Dresden Clinical Institutional Review Board and complies with the Declaration of Helsinki.

#### Dual time point data

Ten patients with different tumor diseases (see Table 1) for which routine dual time point measurements were performed were included (seven males, three females, age 37 to 80 years (mean 60.3), weight 48 to 102 kg (mean 75.2)). For each patient, two whole-body scans were performed with an Ingenuity TF (Philips Healthcare, Best, The Netherlands) time-of-flight PET/MR. The first scan started (78.1 ± 15.9) min (range 58.2 to 110.5 min) after injection of 222 to 306 MBq FDG administrated as bolus over 10 to 20 s. The mean time difference between the first and second scans was 39.2 min (range 24.3 to 55.8). Tomographic images were reconstructed using the BLOB-OS-TF reconstruction (3 iterations, 32 subsets) and MR-based attenuation correction.

**Table 1:** Tumor entities and number of lesions assessed in the dual time point measurements

**Type**	**Number of lesions**
Head and neck cancer	2
Rectal carcinoma (lung metastasis)	4
Rectal carcinoma (liver metastasis)	1
Colon carcinoma (liver metastasis)	1
Colon carcinoma (pelvic metastasis)	1
Non-Hodgkin lymphoma	9
Hodgkin lymphoma	1
Non-small cell lung cancer	2

### Data evaluation

#### Dynamic data

The AIF was determined from a roughly cylindrical 3D region of interest (ROI) centered in the aorta. To exclude partial volume effects, a distance of at least 1 cm from the aortic wall was maintained in all transaxial planes. To compensate for the resulting small transaxial diameter, the ROIs were extended axially (along the aorta) for about 10 cm. This resulted in sufficiently large ROI volumes and ensured sufficiently high statistical accuracy of the derived AIF values. It might be worth to stress that sufficiently accurate determination of the tracer concentration in the arterial blood is crucial in order to obtain reliable SUR values in general and scan-time correction in particular. We consider 2D ROIs in single transaxial slices as insufficient in this context.

Three ‘static’ images were generated from the data acquired from 15 to 25 min (*T*=20 min), 30 to 40 min (*T*=35 min), and 50 to 60 min (*T*=55 min) p.i.. 3D ROIs were defined in 22 lesions in the *T*=55-min image and were transferred to the *T*=20- and *T*=35-min images. The ROIs had volumes from 2.74 to 389 ml (mean 77 ml). Blood concentrations were derived in the same time ranges from the corresponding AIF. For each ROI, the mean SUV and mean SUR were computed at all three time points. The SUR and SUV values of the *T*=20- and *T*=35-min images were then corrected to *T*=55 min using Equations 8 and 10, respectively, and ${\bar{V}}_{r}=0.53$ for the average apparent volume of distribution [7]. The time exponent *b* appearing in Equation 10 was determined from a fit of Equation 5 to the group-averaged AIF and set to the value *b*=0.313. Corrected and uncorrected values were compared with the corresponding values in the *T*=55-min image.

#### Dual time point data

In these investigations, the full dynamic AIF was not accessible, but the blood tracer concentration required for SUR computation at both time points could be determined as follows. For determination of the blood concentration, first, a cylindrical 3D ROI was delineated in the aorta in the MRI scan used for attenuation correction. Here, too, a distance of at least 1 cm from the aortic wall was maintained. The ROI was then transferred to the PET scan, and the blood concentration was computed as the mean value of the ROI. 3D ROIs were defined in 21 lesions in the second DTP image and were transferred to the first one. The ROIs had volumes from 1.26 to 125 ml (mean 24 ml). For each ROI, the mean SUV and mean SUR were computed in both images. The SUR and SUV values of the late scan were then corrected to the time of the early scan, again using Equations 8 and 10, ${\bar{V}}_{r}=0.53$ and *b*=0.313. Corrected and uncorrected values were compared with the corresponding values in the early image.

For, both, dynamic and DTP data absolute and relative differences between uncorrected and corrected SUR values and those measured at the reference time (SUR_ref_) were computed. We use the symbols defined in Table 2, where the reference time *T*=55 min for the dynamic data while for the DTP data it equals the time of the early scan. Differences of SUV values were computed accordingly. Average values are specified as mean ± standard deviation.

**Table 2:** Definition of some symbols (corresponding definitions used for SUV-related quantities)

**Symbol**	**Definition**	**Comment**
SUR_ref_	SUR(*T*_0_)	SUR measured at *t*=*T*_0_ (reference value)
SUR_*T*_	SUR(*T*)^(uncorrected)^	SUR measured at *t*=*T* (without correction to *t*=*T*_0_)
SUR_0_	SUR(*T*)^(corrected)^	SUR measured at *t*=*T* (after correction to *t*=*T*_0_)
*Δ* SUR_*T*_	SUR_*T*_-SUR_ref_	Absolute deviation of uncorrected SUR from reference value
*δ* SUR_*T*_	*Δ* SUR_*T*_/ SUR_ref_	Fractional deviation of uncorrected SUR from reference value
*Δ* SUR_0_	SUR_0_-SUR_ref_	Absolute deviation of corrected SUR from reference value
*δ* SUR_0_	*Δ* SUR_0_/ SUR_ref_	Fractional deviation of corrected SUR from reference value

ROI definition was performed using the ROVER software (ABX, Radeberg, Germany [14,15]). Further data analysis was carried out using the R software for statistical computing [16].

## Results

The time exponent *b* (required to utilize Equation 10 for SUV correction) was set to the value of 0.313, resulting from the hyperbola fit to the AIF data from the dynamic liver metastases investigations (Figure 2). Figure 4 shows the values of *Δ* SUR and *δ* SUR obtained for the dynamic liver metastases data and the two chosen time points *T*=20 min (A, B) and *T*=35 min (C, D). As is to be expected, at both time points, the uncorrected SURs differ quite strongly from the values measured at the reference time *T*=55min: *δ* SUR_*T*_ = (-52 ± 4.5)% at *T*=20 min and *δ* SUR_*T*_ = (-31 ± 3.7)% at *T*=35 min. After correction, the difference is reduced to *δ* SUR_0_ = (-2.9 ± 6.6)% at *T*=20 min and *δ* SUR_0_ = (-2.7 ± 5)% at *T*=35 min. Similar results were found for *Δ* SUV and *δ* SUV (Figure 5). The SUV correction reduces the mean difference from *δ* SUV_*T*_ = (-30 ± 6.6)% to *δ* SUV_0_ = (1.9 ± 6.2)% (*T*=20 min) and from *δ* SUV_*T*_ = (-16 ± 4)% to *δ* SUV_0_ =(1.7 ± 3.3)% (*T*=35 min), respectively. The results for all liver lesions investigated in the dynamic scans are detailed in Tables 3 and 4.

**Figure 4:**
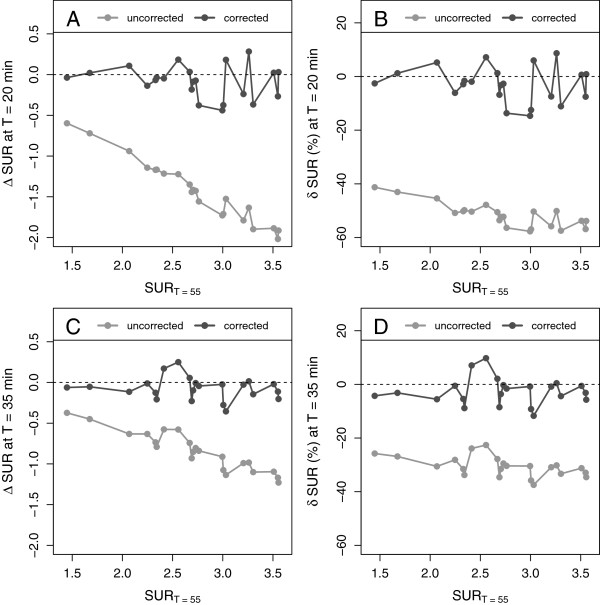
**Absolute and fractional differences of SURs in the dynamic data.** Shown are the differences between uncorrected and corrected SURs, respectively, and the SURs measured at *T*=55 min. **(A)** Absolute difference at *T*=20 min. **(B)** Fractional difference at *T*=20 min. **(C)** Absolute difference at *T*=35 min. **(D)** Fractional difference at *T*=35 min.

**Figure 5:**
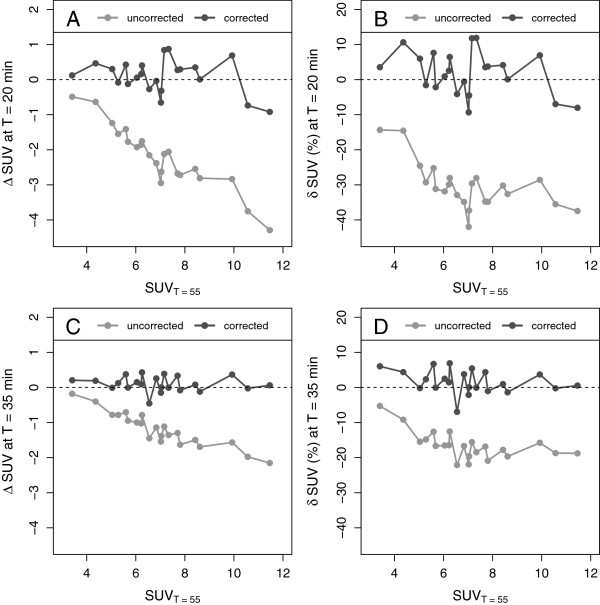
**Absolute and fractional differences of SUVs in the dynamic data.** Shown are the differences between uncorrected and corrected SUVs, respectively, and the SUVs measured at *T*=55 min. **(A)** Absolute difference at *T*=20 min. **(B)** Fractional difference at *T*=20 min. **(C)** Absolute difference at *T*=35 min. **(D)** Fractional difference at *T*=35 min.

**Table 3:** **Dynamic data at *T *** **= 20 min**

	**Lesion number**	**SUR**_***T***_	***δ*** **SUR **_***T***_** (%)**	***δ*** **SUR**_**0**_** (%)**	**SUV **_***T***_	***δ*** **SUV **_***T***_** (%)**	***δ*** **SUV**_**0**_** (%)**
	1	0.9	-41.3	-2.6	2.9	-14.3	3.5
	2	1.0	-43.0	1.2	3.7	-14.6	10.6
	3	1.1	-45.4	5.2	3.8	-24.6	6.0
	4	1.1	-50.8	-6.1	3.7	-29.3	-1.6
	5	1.2	-50.4	-1.9	4.2	-25.2	7.6
	6	1.2	-50.2	-2.9	3.9	-31.2	-2.2
	7	1.3	-56.9	-12.5	4.1	-31.9	0.9
	8	1.2	-53.6	-6.8	4.4	-29.9	2.5
	9	1.3	-52.3	-3.2	4.5	-28.0	6.5
	10	1.2	-49.8	-1.6	4.4	-32.9	-4.1
	11	1.4	-55.8	-7.4	4.5	-34.9	-0.6
	12	1.5	-56.9	-7.5	4.1	-42.0	-9.3
	13	1.4	-57.5	-11.1	4.4	-37.3	-4.5
	14	1.6	-50.1	8.7	5.0	-29.6	11.8
	15	1.5	-50.3	6.0	5.3	-28.0	11.9
	16	1.6	-53.8	0.7	5.0	-34.8	3.5
	17	1.6	-53.8	0.9	5.1	-34.8	3.7
	18	1.3	-50.5	1.3	5.9	-30.2	4.1
	19	1.3	-52.3	-2.7	5.8	-32.6	0.1
	20	1.3	-47.8	7.2	7.1	-28.6	6.9
	21	1.2	-56.4	-13.7	6.8	-35.5	-7.0
	22	1.3	-57.7	-14.7	7.2	-37.4	-8.0
Mean ± SD		1.3±0.2	-51.7±4.5	-2.9±6.6	4.8±1.1	-30.3±6.6	1.9±6.2
Min/max		0.9/1.6	-57.7/-41.3	-14.7/8.7	2.9/7.2	-42.0/-14.3	-9.3/11.9

Absolute values of SUR and SUV and fractional deviations of SUR and SUV from values measured at *T*=55 min for all investigated lesions as well as mean values and range.

**Table 4:** **Dynamic data at *T *** **= 35 min**

	**Lesion number**	**SUR **_***T***_	***δ*** **SUR **_***T***_** (%)**	***δ*** **SUR**_**0**_** (%)**	**SUV **_***T***_	***δ*** **SUV **_***T***_** (%)**	***δ*** **SUV**_**0**_** (%)**
	1	1.1	-25.8	-4.3	3.2	-5.3	6.0
	2	1.2	-26.9	-3.2	4.0	-9.2	4.4
	3	1.4	-30.6	-5.5	4.3	-15.5	-0.2
	4	1.6	-28.1	-0.5	4.5	-14.8	2.3
	5	1.8	-23.9	7.1	4.9	-12.6	6.7
	6	1.6	-31.5	-5.4	4.7	-16.7	-0.1
	7	1.9	-35.8	-9.2	5.0	-16.5	2.5
	8	1.8	-34.6	-8.5	5.2	-16.5	1.5
	9	1.9	-31.5	-3.6	5.5	-12.5	6.9
	10	1.6	-33.8	-8.9	5.1	-22.1	-7.0
	11	2.2	-30.9	-0.8	5.7	-16.7	3.8
	12	2.4	-32.9	-3.2	5.5	-21.9	-2.1
	13	2.2	-33.3	-4.4	5.7	-19.6	0.0
	14	2.3	-30.2	0.4	6.0	-15.6	5.4
	15	1.9	-37.5	-11.7	6.0	-18.5	-0.1
	16	2.4	-31.2	-0.6	6.4	-16.8	4.4
	17	2.3	-34.6	-5.7	6.2	-20.9	-1.0
	18	1.9	-27.8	2.1	6.9	-17.8	1.0
	19	1.9	-29.5	-0.3	6.9	-19.7	-1.4
	20	2.0	-22.6	9.8	8.4	-15.8	3.7
	21	1.9	-30.4	-1.6	8.6	-18.7	-0.2
	22	2.1	-30.4	-0.8	9.3	-18.8	0.6
Mean ± SD		1.9±0.4	-30.6±3.7	-2.7±5.0	5.8±1.5	-16.5±4.0	1.7±3.3
Min/max		1.1/2.4	-37.5/-22.6	-11.7/9.8	3.2/9.3	-22.1/-5.3	-7.0/6.9

Absolute values of SUR and SUV and fractional deviations of SUR and SUV from values measured at *T*=55 min for all investigated lesions as well as mean values and range.

The corresponding results for the DTP data are presented in Figure 6 (SUR) and Figure 7 (SUV). Here, the early time point served as the reference time relative to which the second (late) time point data were corrected. In this case, the correction clearly reduces the differences from *δ* SUR_*T*_ = (48±11)% to *δ* SUR_0_ = (2.6 ± 6.9)% and from *δ* SUV_*T*_ = (24 ± 8.4)% to *δ* SUV_0_ = (-2.4 ± 7.3)%. The results for all lesions investigated in the DTP setup are detailed in Table 5.

**Figure 6:**
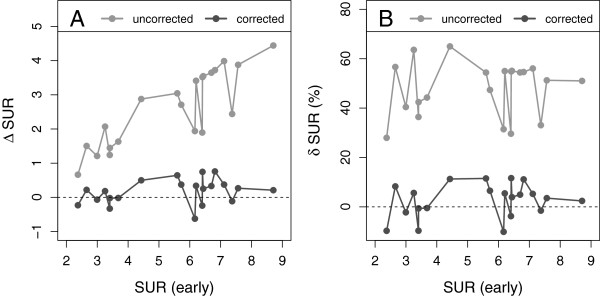
**Differences between uncorrected and corrected late SURs, respectively, and early SURs in the DTP data.(A)** Absolute difference. **(B)** Fractional difference.

**Figure 7:**
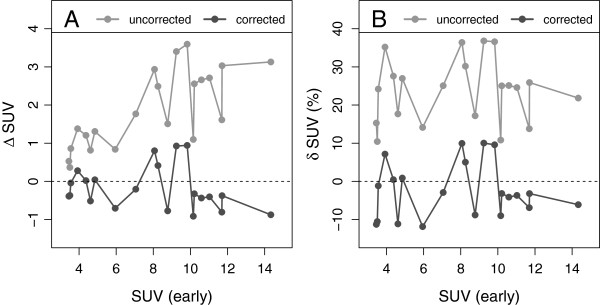
**Differences between uncorrected and corrected late SUVs, respectively, and early SUVs in the DTP data. (A)** Absolute difference. **(B)** Fractional difference.

**Table 5:** DTP data in the late scan

	**Lesion number**	***Δ*** ***T* (min)**	**SUR **_***T***_	***δ*** **SUR **_***T***_** (%)**	***δ*** **SUR**_**0**_** (%)**	**SUV **_***T***_	***δ*** **SUV **_***T***_** (%)**	***δ*** **SUV**_**0**_** (%)**
	1	51.7	4.6	36.4	-9.6	4.0	15.3	-11.3
	2	51.9	3.0	28.0	-9.7	3.9	10.4	-10.5
	3	43.5	4.2	40.4	-2.2	4.4	24.2	-1.2
	4	24.3	4.2	56.7	8.3	5.3	35.2	7.2
	5	24.5	5.3	44.3	-0.5	5.6	27.6	0.5
	6	24.6	5.3	63.6	5.6	5.4	17.7	-11.1
	7	24.6	4.9	42.4	-0.6	6.2	27.0	0.9
	8	39.9	7.3	65.0	11.3	6.8	14.1	-11.9
	9	40.0	11.4	51.3	3.5	8.8	25.1	-2.9
	10	37.6	8.6	54.4	11.5	11.0	36.4	10.0
	11	38.7	8.4	47.4	6.5	10.7	30.2	5.0
	12	34.8	8.1	31.5	-10.1	10.3	17.2	-8.8
	13	34.9	9.9	54.9	11.6	12.6	36.8	10.0
	14	34.9	10.5	54.6	11.1	13.4	36.6	9.6
	15	35.1	8.3	29.6	-3.8	11.3	10.8	-9.0
	16	35.4	9.6	55.0	5.5	12.8	25.0	-3.2
	17	49.7	10.0	55.1	3.9	13.3	25.1	-4.1
	18	55.8	10.3	54.4	5.0	13.7	24.6	-3.7
	19	43.1	9.8	33.1	-1.5	13.3	13.8	-6.9
	20	43.3	11.1	56.1	5.3	14.7	25.9	-3.2
	21	54.6	13.1	51.0	2.4	17.5	21.8	-6.1
Mean ± SD		39.2±9.9	8.0±2.9	47.9±11.0	2.6±6.9	9.8±4.1	23.9±8.4	-2.4±7.3
Min/max		24.3/55.8	3.0/13.1	28.0/65.0	-10.1/11.6	3.9/17.5	10.4/36.8	-11.9/10.0

Absolute values of SUR and SUV and fractional deviations of SUR and SUV from values measured in the early scan for all investigated lesions as well as mean values and range.

## Discussion

Non-negligible variability of scan start relative to the time of injection is a persistent issue in clinical oncological PET [8,9]. In this work, we have developed and investigated a straightforward method to compensate for the spurious changes of SUR and SUV caused by this variability. We want to clarify from the outset that the proposed correction procedure is not intended to obviate the necessity for adequately standardized data acquisition as described, for example, in [17]. Even though the presented scan time correction turns out to work very well even for substantial deviations from the targeted reference scan time, it is of course preferable to minimize the influence of the correction (with its inherent remaining uncertainties) by adhering to a standardized time p.i. as closely as practically possible.

The method was evaluated in two patient groups, first, in 22 liver lesions (15 scans) measured dynamically for 60 min and furthermore in 21 lesions (10 scans) assessed with DTP measurements. For each investigation, a reference time was selected from the available time frames (midpoint of the last time frame (*T*=55 min) for the dynamic studies and of the early DTP time frame (78.1 ± 15.9 min), respectively). SUR and SUV measured at the respective reference time were used as the ‘gold standard’ against which the corrected uptake values acquired at further time points were compared.

The main finding of this work is that correction for scan time variability (more precisely, correction for the accompanying spurious SUR and SUV changes) is feasible with surprisingly good accuracy and does only require an image-based determination of the arterial tracer concentration at the given scan time (rather than knowledge of the full AIF): group averaged differences between corrected and reference values typically only amount to about (3±7)% (or even less) and maximal deviations remain below ±15% in all cases. This is to be compared with group averaged deviations of up to about 50% (and maximal deviations of 65%) without correction.

Especially, the obtained accuracy can be considered sufficient to allow reliable comparison of lesion SURs and SUVs in follow-up studies during therapy response assessment, where typical inter-scan changes of 50% are considered as relevant (see e.g., [18] and references therein) even if the scan time varies significantly between the successive scans. Moreover, compensation for scan time variability does offer the possibility to investigate whether smaller SUV changes (currently masked by scan time variability effects) could also provide relevant diagnostic information.

Two reasons can be identified for the observed residual differences *Δ* SUR_0_=SUR_0_-SUR_ref_ between SUR measured at time *T* (and corrected to chosen reference time) and the SUR actually measured at that reference time. The first reason, obviously, are limitations of the correction procedure and its underlying assumptions. The second one is the fact that we use SUR_ref_ as our *de facto* ‘gold standard’ since the real ground truth SUR at that time point is not known. Therefore, the resulting *Δ* SUR_0_ is affected by statistical and systematic errors of both measured SUR values (SUR_0_ and SUR_ref_). In order to estimate the relative influence of both factors, we tentatively eliminated the first one by using Equation 4 instead of Equation 8 for correction and re-evaluation of the dynamic data using the individually correct *V*_r_ resulting from Patlak analysis and the actual *Θ*_*T*_ resulting from integration of the individually measured dynamic AIF up to time *T*. This procedure reduces the correction to application of the individually correct Patlak plot which thus would be ‘exact’ if the individual dynamic data points were perfectly following the Patlak equation. For *T*=20 min, we find a reduction of the mean difference of SUR from (-2.9±6.6)% to (1.52±3.8)%, i.e., the mean value and the standard deviation of the difference are reduced by about 50%. The still remaining deviations can be attributed to the statistical and systematic errors of the two SUR measurements contributing to the correction. Therefore, it might be concluded that the accuracy of the presented scan time correction method actually is roughly twice as high than our results initially suggest: its inherent uncertainty is comparable to the statistical accuracy of the measured SUV and SUR values themselves.

The presented method rests on the empirical fact that the AIF after FDG bolus injection exhibits an invariant shape that, moreover, leads to an approximate proportionality between Patlak time *Θ* and actual scan time *T* (see Figure 1). This observation is mathematically equivalent to stating that starting very early after injection, the AIF can be described by a simple power law (see Figure 2). This specific, investigation-independent *Θ*(*T*) relation (Equation 7) ultimately leads to Equation 8. Thus, SUR correction back to the actually intended (reference) scan time only requires determination of the respective SUR, i.e., of tissue SUV as well as AIF level at the given scan time. It is then computable from the deviation of the given from the intended start time alone (using an estimate of the small parameter ${\bar{V}}_{r}$ whose precise value is not critical for the correction). An interesting observation in this context is the fact that the actual value of the time exponent *b* in the power law used for describing the AIF does not explicitly enter the SUR correction (Equation 8) but only the SUV correction formula (Equation 10). The required value of *b* was determined by a least squares fit of Equation 5 to the available group averaged dynamic AIF (see Figure 2) resulting in *b*=0.313. Using this value of *b* yielded a correction of SUV with an accuracy comparable to that of the SUR correction.

Strictly speaking, our dynamic liver scans only demonstrate that the performed parametrization of the AIF shape by an inverse power law and the stated value of the parameter *b* holds up to about 60 min p.i.. Beyond that time range, no direct proof for this specific (and invariant) shape exists. However, the very good performance of the correction procedure not only for the dynamic liver metastases data measured up to *T*=60 min but also for the DTP measurements (where the second scan occurred on average about 2 h p.i.) provides strong evidence that Equation 7 can be extrapolated to distinctly later times (with the same value for *b*) without causing notable errors in the correction procedure. Moreover, the SUR correction (Equation 8) is not affected by the actual value of *b* (and would be unaltered even in a constant infusion scenario corresponding to *b*=0), while the SUV correction (Equation 10) is not critically sensitive to modest variations of *b*. Good performance of the correction in the DTP group furthermore shows that the chosen value ${\bar{V}}_{r}=0.53$ (also derived from the dynamic AIF data of the liver metastases investigations) is applicable for all tumor entities included in the DTP investigations.

The chosen parametrization of the AIF by a power law allows a concise formulation of the correction formulas. While this parametrization is not usually applied for empirical modelling of AIF shapes (where sums of exponentials are more common), it is not only justified by our results but also consistent with the published data of Thie et al. which modeled *Θ*(*T*) (*T*_eq_(*t*) in their notation) as a third-order polynomial (Equation (3) in [10]). In the time range used by Thie et al. (up to 60 min p.i.), the non-linearity is very small and their relation quite well described by *Θ*(*T*)=1.67×*T* which deviates by less than 15% from our result *m*=1/(1-*b*)=1.46. While the power law approach thus seems suggested by the data and is attractive due to its dependence on only two free parameters, we would like to emphasize that a different AIF modelling would also be possible and would yield comparable results regarding accuracy of the correction, presuming the parametrization is adequately fitting the measured AIF data. For example, Vriens and coworkers used three exponentials to model a population averaged AIF after bolus transition up to *T*=45 min [19]. Fitting the power law in Equation 5 to their parameterized AIF leads to *b*≈0.34 which also is consistent with our result *b*=0.313±0.030. Moreover, as already pointed out, adjusting the value of *b* only affects (slightly) the SUV correction, but not the SUR correction.

Notable differences are to be expected, however, if different parameterizations of the AIF are extrapolated beyond the time range used in the fitting procedure. Especially, an extrapolated exponential model (derived from the limited early time range of dynamic measurements) will rapidly decrease to negligible concentration levels at later times which is not in accord with reality.

On the other hand, our extrapolation of the power law to the much later times used in the DTP measurements could be proven to yield very satisfactory results regarding SUR/SUV correction. Therefore, our assumption is that a power law with *b*≈0.3 remains valid for AIF modelling also at late times. The good performance of the correction procedure for the DTP data (acquired with a different scanner at much later times and also reconstructed differently) provides strong evidence for this conjecture. Despite these promising results, further investigations are desirable to further support the assumed power law behavior of the AIF at later times.

Ultimately, the proposed SUR (and SUV) correction rests on the ability to derive the lesion’s *K*_m_ from its measured SUR according to Equation 3. As already stated in [7] this is not necessarily always correct, e.g., if inflammation is involved, this assumption no longer hold since the Patlak assumption of irreversible trapping is violated in this case. For that reason, we did not recommend a general conversion from SUR to *K*_m_ in [7]. One example might be the finding in [11] that a fraction of breast cancer lesions exhibited untypical time dependence of lesion SUV (very low and either essentially time independent or even slightly decreasing). While such behavior (including slightly decreasing SUV over time) still is compatible with irreversible kinetics if *K*_m_ is sufficiently small (due to the then dominant contribution of the reversible FDG pool to the PET signal which follows the decrease of the AIF), it also might be caused by actual deviations from irreversible kinetics. From a practical point of view, as far as lesions with a very low SUV are concerned (as was the case in [11]), such deviations from irreversible transport have no real impact on the scan time correction for the simple reason that the absolute magnitude of the correction is small if the SUV itself is small, so, even if the correction would be erroneous in this situation, it would simply represent a small (erroneous) correction of a small SUV with no further practical consequences. We reiterate, however, that the correction procedure rests on the assumption of irreversible tracer kinetics and might lead to erroneous conclusions if this assumption is violated. On the other hand, the assumption has turned out to be valid for all tumor lesions we have investigated so far (notably the quite heterogeneous group of tumor entities present in our DTP data).

Altogether, scan time correction of SUR thus seems feasible whenever the tracer kinetics can be adequately described by a Patlak equation. Scan time correction of SUV is then possible as well, but somewhat more dependent on the applied AIF parametrization (explicit dependence on *b*). We believe it also would be worthwhile to investigate whether the procedure could be extended to other mostly irreversibly binding PET tracers (i.e., tracers for which influence of a possibly non-zero *k*_4_ rate constant in the standard reversible two-compartment model is negligible in the considered time range) if SUV-based approaches are applied since they would be affected by scan time variability in a comparable way.

Finally, it is important to point out the fact that the time dependency of SUR is distinctly larger than that of SUV which makes scan time correction even more important for the former. The increased time dependence is easily explained by observing that SUR is defined as the ratio of (increasing) SUV and (decreasing) arterial blood concentration. For our data, the difference between SUR at the actual scan time *T* and SUR at the reference time was up to *δ* SUR_*T*_≈60*%*. The SUV difference was lower, up to *δ* SUV_*T*_≈40*%*, but still far from negligible. Moreover, the scan time correction of SUV does of course not reduce the independent substantial influence of inter-study variability in blood concentration on the resulting SUV [7]. This distinct disadvantage of SUV relative to SUR persists, and we believe that a transition from SUV-based (usually corrected neither for scan time nor for arterial blood concentration variability) to SUR-based evaluation (including correction for scan time variability as proposed in the present work) could offer distinct advantages for quantitative oncological FDG PET.

## Conclusion

If FDG kinetics is irreversible in the targeted tissue, correction of standard uptake values and tumor-to-blood uptake ratios for scan time variability is possible with good accuracy. The correction distinctly improves comparability of lesion uptake values measured at different times post injection.

## Competing interests

The authors declare that they have no competing interests.

## Authors’ contributions

JVDH had the initial idea for time correction of SUR and SUV, performed part of the data analysis, and wrote part of the manuscript. AL and GS performed part of the data analysis. JM, LO, JP, and JK provided intellectual input and reviewed the manuscript. BBB selected the patient studies and performed the lesion delineation. FH performed part of the data analysis and wrote part of the manuscript. All authors read and approved the final manuscript.
